# Synergistic Roles of Eukaryotic Translation Elongation Factors 1Bγ and 1A in Stimulation of Tombusvirus Minus-Strand Synthesis

**DOI:** 10.1371/journal.ppat.1002438

**Published:** 2011-12-15

**Authors:** Zsuzsanna Sasvari, Lara Izotova, Terri Goss Kinzy, Peter D. Nagy

**Affiliations:** 1 Department of Plant Pathology, University of Kentucky, Lexington, Kentucky, United States of America; 2 Department of Molecular Genetics, Microbiology, and Immunology, University of Medicine and Dentistry of New Jersey Robert Wood Johnson Medical School, Piscataway, New Jersey, United States of America; University of California, Riverside, United States of America

## Abstract

Host factors are recruited into viral replicase complexes to aid replication of plus-strand RNA viruses. In this paper, we show that deletion of eukaryotic translation elongation factor 1Bgamma (eEF1Bγ) reduces *Tomato bushy stunt virus* (TBSV) replication in yeast host. Also, knock down of eEF1Bγ level in plant host decreases TBSV accumulation. eEF1Bγ binds to the viral RNA and is one of the resident host proteins in the tombusvirus replicase complex. Additional *in vitro* assays with whole cell extracts prepared from yeast strains lacking eEF1Bγ demonstrated its role in minus-strand synthesis by opening of the structured 3′ end of the viral RNA and reducing the possibility of re-utilization of (+)-strand templates for repeated (-)-strand synthesis within the replicase. We also show that eEF1Bγ plays a synergistic role with eukaryotic translation elongation factor 1A in tombusvirus replication, possibly via stimulation of the proper positioning of the viral RNA-dependent RNA polymerase over the promoter region in the viral RNA template.These roles for translation factors during TBSV replication are separate from their canonical roles in host and viral protein translation.

## Author Summary

RNA viruses recruit numerous host proteins to facilitate their replication and spread. Among the identified host proteins are RNA-binding proteins (RBPs), such as ribosomal proteins, translation factors and RNA-modifying enzymes. In this paper, the authors show that deletion of eukaryotic translation elongation factor 1Bgamma (eEF1Bγ) reduces *Tomato bushy stunt virus* (TBSV) replication in a yeast model host. Knock down of eEF1Bγ level in plant host also decreases TBSV accumulation. Moreover, the authors demonstrate that eEF1Bγ binds to the viral RNA and is present in the tombusvirus replicase complex. Functional studies revealed that eEF1Bγ promotes minus-strand synthesis by serving as an RNA chaperone. The authors also show that eEF1Bγ and eukaryotic translation elongation factor 1A, another host factor, function together to promote tombusvirus replication.

## Introduction

Plus-stranded (+)RNA viruses recruit numerous host proteins to facilitate their replication and spread [1], [2]. Among the identified host proteins are RNA-binding proteins (RBPs), such as ribosomal proteins, translation factors and RNA-modifying enzymes [1]–[5]. The subverted host proteins likely affect several steps in viral RNA replication, including the assembly of the replicase complex and initiation of RNA synthesis. However, the detailed functions of recruited host RBPs in (+)RNA virus replication are known only for a small number of host factors [2], [6]–[8].

*Tomato bushy stunt virus* (TBSV) is model plant RNA virus coding for two replication proteins, p33 and p92^pol^, which are sufficient to support TBSV replicon (rep)RNA replication in a yeast (*Saccharomyces cerevisiae*) model host [9], [10]. p33 and p92^pol^ are components of the membrane-bound viral replicase complex, which also contains the tombusviral repRNA serving not only as a template for replication, but also as a platform for the assembly of the viral replicase complex [11]–[13]. Recent genome-wide screens and global proteomics approaches with TBSV and a yeast host revealed a large number of host factors interacting with viral components or affecting TBSV replication. The identified host proteins are involved in various cellular processes, such as translation, RNA metabolism, protein modifications and intracellular transport or membrane modifications [14]–[17].

Various proteomics analyses of the highly purified tombusvirus replicase has revealed at least five permanent resident host proteins in the complex, including the heat shock protein 70 chaperones (Hsp70) [18]–[21], glyceraldehyde-3-phosphate dehydrogenase [4], pyruvate decarboxylase [21], Cdc34p E2 ubiquitin conjugating enzyme [4], [21], [22], eukaryotic translation elongation factor 1A (eEF1A) [23], [24] and two temporary resident proteins, Pex19p shuttle protein [25] and the Vps23p adaptor ESCRT protein [24], [26], [27]. The functions of several of these proteins have been studied in some detail [4], [17], [18], [19], [20].

The emerging picture from systems biology approaches is that eukaroyotic translation elongation factors (eEFs), such as eEF1A, play several roles during TBSV replication. Accordingly, eEF1A has been shown to facilitate the assembly of the viral replicase complex and stimulate the initiation of minus-strand synthesis by the viral RNA-dependent RNA polymerase (RdRp) [23], [24]. Another translation elongation factor identified in our genome-wide screens with TBSV is eukaryotic elongation factor 1Bgamma (eEF1Bγ) [15]. eEF1Bγ is an abundant, but not essential cellular protein, which is part of the eukaryotic translation elongation factor 1B complex also containing the eEF1Bα subunit in yeast and the eEF1Bα and eEF1Bδ subunits in metazoans [28].The eEF1B complex is the guanine nucleotide exchange factor for eEF1A, which binds and delivers aminoacyl-tRNA in the GTP-bound form to the elongating ribosome. Additional roles have been ascribed to eEF1Bγ in vesicle-mediated intracellular protein transport, RNA-binding, vacuolar protein degradation, oxidative stress, intermediate filament interactions and calcium-dependent membrane-binding [29], [30], [31].

In this paper, we characterize the function of eEF1Bγ in TBSV replication. Our approaches based on yeast and *in vitro* replication assays reveal that eEF1Bγ is a component of the tombusvirus replicase and binds to the 3′-end of the viral RNA. Using a cell-free replication assay, we define that eEF1Bγ plays a role by enhancing minus-strand synthesis by the viral replicase. The obtained data support the model that eEF1Bγ opens up a ‘closed’ structure at the 3′-end of the TBSV (+)RNA, rendering the RNA compatible for initiation of (-)-strand synthesis. Moreover, we find that eEF1Bγ and eEF1A play nonoverlapping functions to enhance (-)-strand synthesis. Altogether, the two translation factors regulate TBSV replication synergistically by interacting with different portions of the viral (+)RNA and the replication proteins.

## Results

### Deletion of eEF1Bγ inhibits TBSV RNA accumulation in yeast model host

**Figure 1:**
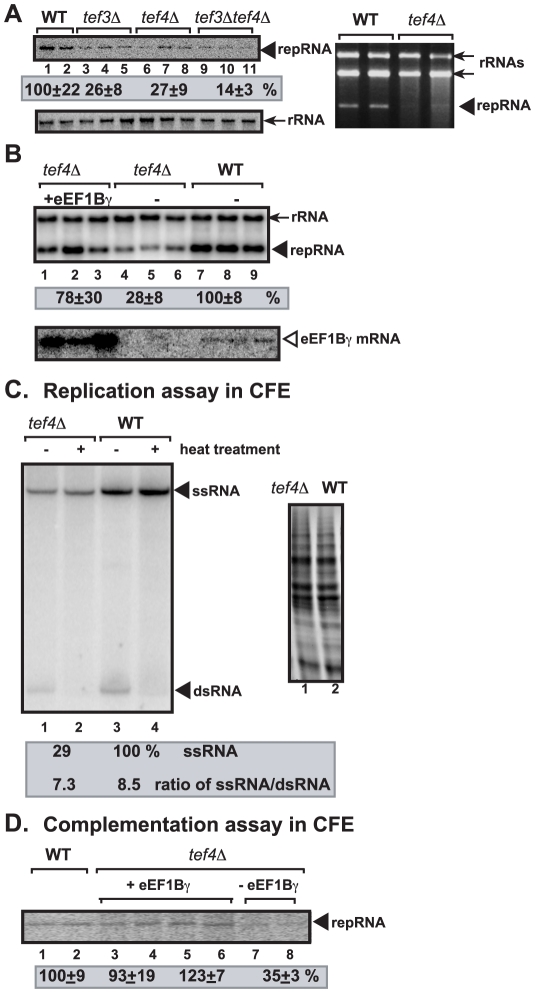
The effect of deletion of the *TEF3* and *TEF4* yeast genes coding for eEF1Bγ on TBSV repRNA accumulation in yeast and in a cell-free extract. (A) Top left panel: Replication of the TBSV repRNA was measured by Northern blotting 24 h after initiation of TBSV replication in the shown yeast strains. The accumulation level of repRNA was normalized based on the rRNA (middle panel, the 18S ribosomal RNA levels were estimated by Northern blotting). Each sample was obtained from different yeast colonies. Top right panel: Ethidium-bromide stained agarose gel shows the reduced accumulation of TBSV repRNA in *tef4*Δ yeast. (B) Complementation of *tef4*Δ yeast with plasmid-based Tef4p (eEF1Bγ). The expression of the *TEF4* mRNA is shown in the bottom panel based on Northern blotting. (C) Cell-free TBSV replicase assay supports a role for eEF1Bγ in minus-strand synthesis. Purified recombinant TBSV p33 (12 pmol) and p92^pol^ (1 pmol) replication proteins in combination with DI-72 (+)repRNA (4 pmol)were added to the whole cell extract prepared from *tef4*Δ (lanes 1–2) or WT yeast strains. Left panel: The nondenaturing PAGE analysis of the ^32^P-labeled repRNA products obtained is shown. The full-length single-stranded repRNA is pointed at by an arrow. Odd numbered lanes represent replicase products, which were not heat treated (thus both ssRNA and dsRNA products are present), while the even numbered lanes show the heat-treated replicase products (ssRNA is present). The amount of ssRNA and the ratio of ssRNA/dsRNA in the samples are shown. Note that, in the nondenatured samples, the dsRNA product represents the annealed (-)RNA and the input (+)RNA, while the ssRNA products represents the newly made (+)RNA products. Right panel shows the coomassie-blue stained SDS-PAGE gel to visualize total protein levels in the whole cell extracts. (D) eEF1Bγ stimulates TBSV repRNA synthesis in whole cell extract prepared from *tef4*Δ. Increasing amounts of purified recombinant eEF1Bγ (lanes 3–4, 26 pmol; lanes 5–6, 13 pmol) were added to *tef4*Δ CFE and the *in vitro* synthesized ^32^P-labeled TBSV repRNA was measured on denaturing PAGE. See further details in panel C. Note that the recombinant eEF1Bγ added to the *tef4*Δ CFE is about 10-fold less than the total eEF1Bγ present in the WT CFE.

eEF1Bγ is coded by *TEF3* and *TEF4* nonessential genes in yeast [32], [33]. Single deletion of *TEF3(CAM1)* or *TEF4* reduced TBSV repRNA accumulation to ∼25% (Figure 1A, lanes 3–8), while deletion of both genes resulted in even more inhibition, supporting TBSV repRNA accumulation only at 15% level (lanes 9–11). Expression of eEF1Bγ (Tef4p) in *tef4*Δ yeast increased TBSV replication to ∼80%, demonstrating that the defect in TBSV repRNA replication in *tef4*Δ yeast can be complemented.Altogether, these data established that eEF1Bγ plays an important stimulatory role in TBSV replication.

### Depletion of eEF1Bγ inhibits (-)-strand synthesis by the TBSV replicase in a cell-free extract

To obtain direct evidence on the involvement of eEF1Bγ in TBSV replication, we prepared cell-free extracts (CFE) from a yeast strain lacking the *TEF4* gene or from wt yeast. These yeast extracts contained comparable amount of total proteins (Figure 1C, right panel). The CFE extracts were programmed with the TBSV (+)repRNA and purified recombinant p33 and p92^pol^ obtained from *E. coli.* Under these conditions, the CFE supports the *in vitro* assembly of the viral replicase, followed by a single cycle of complete TBSV replication, resulting in both (-)-stranded repRNA and excess amount of (+)-stranded progeny [20], [34]. Importantly in the case of a translation factor, this assay uncouples the translation of the viral proteins from viral replication, which are interdependent during (+)RNA virus infections.

CFE obtained from *tef4*Δ yeast supported only 29% of TBSV repRNA replication when compared with the extract obtained from wt yeast (Figure 1C, lane 2 versus 4). These data demonstrate that Tef4p plays an important role in the activity of the viral replicase complex.

To test if the decrease in TBSV repRNA replication *in vitro* was due to reduced (+) or (-)-strand synthesis, we measured the replication products under non-denaturing versus denaturing conditions (Figure 1C). We found that the amount of dsRNA [representing the newly-synthesized ^32^P-labeled (-)RNA product hybridized with the input (+)RNA; lane 1, Figure 1C, see also ref. [23]] and the newly-synthesized (+)RNA both decreased by ∼3-fold in CFE obtained from *tef4*Δ yeast in comparison with those products in the wt CFE (lane 3). Since the ratio of dsRNA and ssRNA did not change much in the CFEs (Figure 1C), the obtained data are consistent with the model that Tef4p (eEF1Bγ) affects the level of (-)RNA production, which then leads to proportionately lower level of (+)RNA progeny.

Adding purified recombinant eEF1Bγ to CFE from *tef4*Δ yeast supported TBSV repRNA replication to similar extent as the CFE from wt yeast (i.e., containing wt eEF1Bγ, Figure 1D, lanes 3–6 versus 1–2), indicating that the recombinant eEF1Bγ can complement the missing Tef4p *in vitro*, when the same amount of p33 and p92^pol^ was provided. Using large amount of eEF1Bγ in the CFE-based assay did not further increase TBSV repRNA replication (Figure 1D, lanes 3–4), suggesting that eEF1Bγ should be present in optimal amount during TBSV replication.

### eEF1Bγ stimulates initiation of (-)RNA synthesis by a viral RdRp *in vitro*

**Figure 2:**
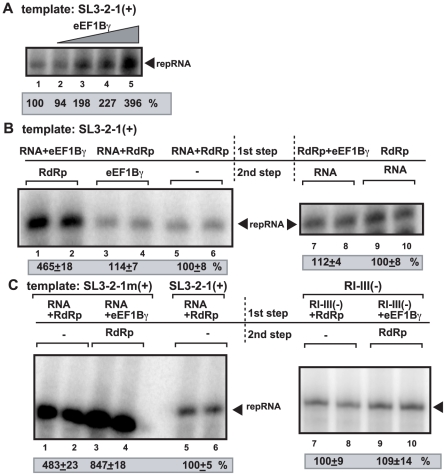
eEF1Bγ promotes minus-strand synthesis by the closely-related TCV RdRp. (A) Purified eEF1Bγ was added to the TCV RdRp assay as shown. The TBSV (+)RNA template was the short 3′ end region [SL3-2-1(+), 20 pmol], which contain the promoter region (SL1) for initiation and the replication silencer element (within SL3) that down-regulates initiation. The gel image shows the results of RNA synthesis in a TCV RdRp assay in the presence of 2, 10, 20 and 40 pmol eEF1Bγ. 2 pmol of purified TCV RdRp was used in these assays. (B) The TCV RdRp assay had two steps: first, the shown components were incubated at room temperature to facilitate their interaction, followed 5 min later the addition of the shown component and the ribonucleotides to start RNA synthesis. The RdRp activity in samples containing the template RNA and the RdRp were chosen as 100% (lanes 5–6 and 9–10). The RNA transcript (20 pmol), eEF1Bγ (20 pmol) and purified TCV RdRp (2 pmol) were used in these assays. (C) The effect of eEF1Bγ on the TCV RdRp activity with additional templates. One of the templates was SL3-2-1 m(+) with a point mutation within the promoter sequence (carrying SL1m mutation), which is being used more efficiently than the wt SL3-2-1(+) by the TCV RdRp *in vitro*. The second template was RI-III(-) representing portions of the minus-stranded RNA. See further details in panel B.

To obtain additional evidence if eEF1Bγ could stimulate RNA synthesis by the viral RdRp, we used the *E. coli*-expressed recombinant p88C^pol^ RdRp protein of *Turnip crinkle virus* (TCV). The TCV RdRp, unlike the *E. coli*-expressed TBSV p92^pol^ or the closely-related *Cucumber necrosis virus* (CNV) p92^pol^ RdRps, does not need the yeast CFE to be functional *in vitro* [35], [36]. Importantly, the template specificity of the recombinant TCV RdRp with TBSV RNAs is similar to the closely-related tombusvirus replicase purified from yeast or infected plants [10], [36], [37], [38]. The recombinant TCV RdRp preparation lacks co-purified eEF1Bγ (*E. coli* does not have a homolog), unlike the yeast or plant-derived tombusvirus replicase preparations, facilitating studies on the role of eEF1Bγ on the template activity of a viral RdRp. When we added various amounts of the highly purified recombinant eEF1Bγ to the TCV RdRp assay programmed with TBSV-derived SL3-2-1(+) RNA template, which is used by the TCV RdRp *in vitro* to produce the complementary (-)RNA product [37], we observed a ∼2-to-4-fold increase in (-)RNA synthesis by the TCV RdRp (Figure 2A, lanes 3–5). eEF1Bγ in the absence of the TCV RdRp did not give a ^32^P-labeled RNA product, excluding that our eEF1Bγ preparation contained RdRp activity (not shown). Altogether, our data suggest that eEF1Bγ can stimulate *in vitro* activity of TCV RdRp on a TBSV (+)RNA template, confirming a direct role for eEF1Bγ in viral (-)RNA synthesis by a viral RdRp.

To test if the stimulating activity of eEF1Bγ on the *in vitro* RdRp activity was due to binding of eEF1Bγ to the (+)RNA template and/or to the TCV RdRp protein, we performed assays, in which the recombinant eEF1Bγ was pre-incubated with the TCV RdRp or the (+)RNA template prior to the RdRp assay. These experiments revealed that pre-incubation of the purified eEF1Bγ with the TBSV-derived SL3-2-1(+) RNA template prior to the RdRp assay led to a ∼4.5-fold increase in (-)RNA products (Figure 2B, lanes 1–2). In contrast, pre-incubation of the TCV RdRp with the (+)RNA template (Figure 2B, lanes 3–4) or eEF1Bγ with the TCV RdRp (Figure 2B, lanes 7–8) prior to the RdRp assay did not result in increase in (-)RNA synthesis. Overall, data shown in Figure 2B imply that eEF1Bγ can stimulate (-)RNA synthesis only when eEF1Bγ binds to the (+)RNA template before the RdRp binding to the template.

To further test the stimulatory effect of eEF1Bγ, we also tested the RdRp activity in the presence of eEF1Bγ using a mutated (+)RNA template. The mutation [SL3-2-1m(+)] opens up the closed structure in the promoter region that leads to increased template activity [39]. The mutated template showed only ∼2-fold increased RNA products in the RdRp assay with eEF1Bγ (Figure 2C, lanes 3–4 versus 1–2). In contrast, eEF1Bγ did not stimulate RNA products when the negative-stranded RI-III(-) RNA was used as a template in the TCV RdRp assay (Figure 2C, lanes 9–10 versus 7–8). Thus, these data support the model that eEF1Bγ can mainly stimulate (-)-strand synthesis by the RdRp on the wt 3′ TBSV sequence, while it is not effective on the (-)RNA template.

### eEF1Bγ binds to the 3′ end of the TBSV RNA *in vitro*

To test if eEF1Bγ directly binds to a particular region within the TBSV repRNA, we performed electrophoretic mobility shift (EMSA) experiments with purified eEF1Bγ and ^32^P-labeled regions of (+)repRNA that included known cis-acting elements involved in (-)RNA synthesis [39], [40], [41]. These experiments revealed that eEF1Bγ bound efficiently to the 3′-end of the TBSV (+)repRNA (construct SL3-2-1, carrying the terminal 3 stem-loop structures, Figure S1). Template competition experiments confirmed that SL3-2-1 RNA bound competitively to eEF1Bγ *in vitro*(Figure S1B).

**Figure 3:**
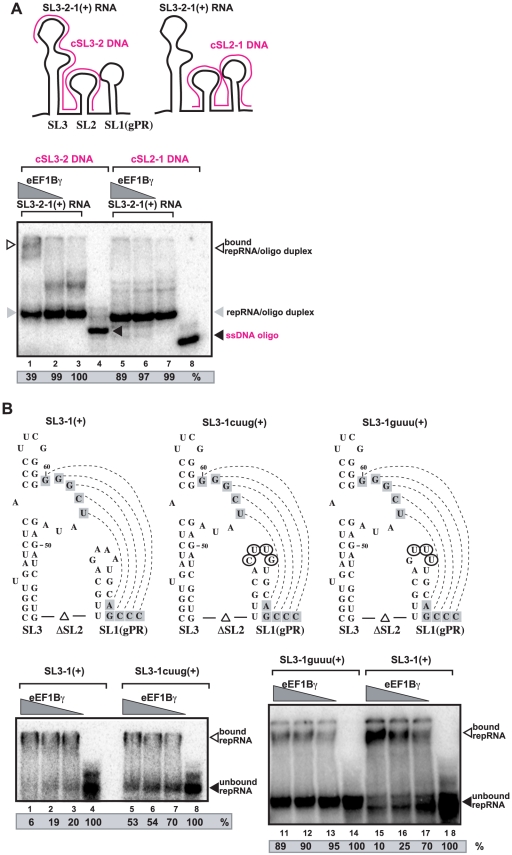
eEF1Bγ binds to the 3′ end of the TBSV (+)RNA. (A) *in vitro* binding assay with purified eEF1Bγ using an ssDNA oligo/ssRNA template duplex. The annealed ssDNA (purple)/ssRNA (black) duplexes representing the 3′ end of the TBSV RNA are shown schematically. The assay contained the annealed ssDNA/ssRNA plus 0.6 and 0.4 pmol purified recombinant eEF1Bγ, respectively. The ^32^P-labeled free ssDNA and ssDNA/ssRNA duplex were separated on nondenaturing 5% acrylamide gels. Quantification of the ssDNA/ssRNA duplex was done with ImageQuant. (B) RNA gel shift analysis shows the role of the SL1 tetraloop in binding to eEF1Bγ. The RNA templates representing the 3′ end of the TBSV RNA and the mutations (circled nucleotides) are shown schematically. The eEF1Bγ - ^32^P-labeled ssRNA complex was visualized on nondenaturing 5% acrylamide gels. The RNA transcript (0.2 pmol), and eEF1Bγ (0.4, 0.5 and 0.6 pmol) were used in these assays.

To further define what sequence within SL3-2-1 is bound by eEF1Bγ, we used complementary DNA oligos to partially convert portions of SL3-2-1 into duplexes (RNA/DNA hybrids) as shown in Figure 3A. EMSA assay with purified recombinant eEF1Bγ revealed that the very 3′-terminal SL1 region had to be “free” (not part of the duplex) for eEF1Bγ to bind efficiently to the SL3-2-1 RNA (compare lane 1 with lane 5 in Figure 3A).

Since eEF1Bγ is known to bind to A-rich single-stranded sequences [32], we mutagenized the tetraloop (GAAA) sequence to either CUUG or GUUU tetraloop sequences (Figure 3B) that are expected to maintain the stability of the double-stranded stem. EMSA analysis showed that neither RNAs with the new tetraloop sequences bound efficiently to eEF1Bγ (Figure 3B, lanes 5–7 and 11–13). Based on the EMSA data, we conclude that the GAAA tetraloop region of SL1 is an efficient binding site for eEF1Bγ *in vitro*. However, we cannot exclude that eEF1Bγ binding may be dependent on stabilizing effects of the GNRA tetraloop on the stem structure. The loop nucleotides may or may not be involved in protein-RNA contacts.

### Binding of eEF1Bγ to the 3′ end of the TBSV RNA is required for stimulation of (-)-strand RNA synthesis *in vitro*

**Figure 4:**
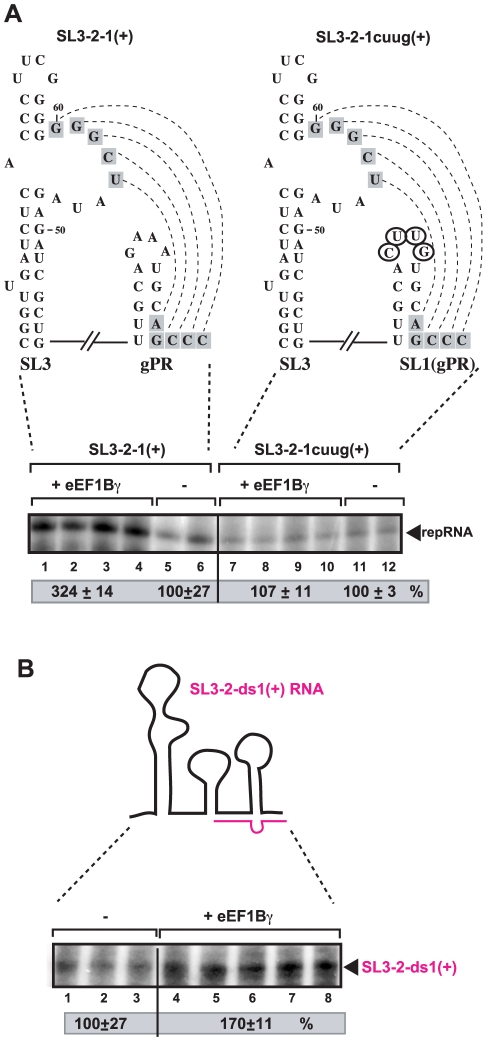
eEF1Bγ stimulates the RdRp activity of a viral polymerase on a TBSV template *in vitro*. (A) Schematic presentation of the RNA templates representing the wt and mutated 3′ ends of TBSV (+)RNA. The middle-range RSE-gPR interaction is shown with dotted lines and the nucleotides involved are in gray boxes. The mutated nucleotides are encircled, while the SL2 hairpin is not shown. The panel shows a representative denaturing gel of ^32^P-labeled RNA product synthesized by TCV p88C RdRp *in vitro* in the presence of 20 pmol of purified recombinant eEF1Bγ. The level of RNA synthesis was compared to that of the RdRp activity obtained in the absence of eEF1Bγ (100%). Each experiment was repeated three times. (B) eEF1Bγstimulates the RdRp activity of a viral polymerase *in vitro* on a duplex (partially double-stranded) RNA template. *In vitro* RdRp assay was performed with TCV p88C (2 pmol) in the presence or absence of purified eEF1Bγ (20 pmol) using a partial dsRNA template (20 pmol, as shown schematically on the top(. The level of RNA synthesis was compared to that of the RdRp activity obtained in the absence of eEF1Bγ (100%). (Each experiment was repeated three times. (B) eEF1Bγstimulates the RdRp activity of a viral polymerase in vitro on a duplex (partially double-stranded) RNA template. In vitro RdRp assay was performed with TCV p88C (20 pmol( in the presence or absence of purified eEF1Bγ (20 pmol( using a partial dsRNA template (20 pmol, as shown schematically on the top(. The level of RNA synthesis was compared to that of the RdRp activity obtained in the absence of eEF1Bγ (100%).

To examine if binding of eEF1Bγ to SL1 is important for stimulation of (-)-strand RNA synthesis by the viral RdRp, we performed an *in vitro* RNA synthesis assay using a mutated SL3-2-1 carrying the ‘CUUG’ tetraloop instead of the wt ‘GAAA’ tetraloop sequence (Figure 4A). Unlike for the wt SL3-2-1 RNA, eEF1Bγ could not stimulate complementary RNA synthesis by the viral RdRp on the SL3-2-1cuug(+) template (Figure 4A, lanes 7–10 versus 1–4). These data suggest that binding of eEF1Bγ to the ‘GAAA’ tetraloop sequence of SL1 is important to stimulate (-)-strand synthesis by the viral RdRp *in vitro*.

### eEF1Bγ co-purifies with the viral replicase complex and it binds to TBSV repRNA in yeast

**Figure 5:**
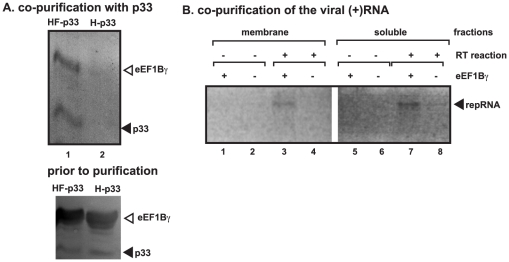
Co-purification of eEF1Bγ with the p33 replication protein and the viral RNA from yeast. (A) Top panel: Western blot analysis of p33 with the co-purified eEF1Bγ protein. The FLAG/His_6_-tagged HF-p33 was purified from yeast extracts using a FLAG-affinity column. The purified HF-p33 and the co-purified His_6_-tagged eEF1Bγ were detected with anti-His antibody. Bottom panel: Western blot of HF-p33 and the His_6_-tagged eEF1Bγ in the total yeast extract using anti-His antibody. (B) RT-PCR analysis to detect the co-purified TBSV (+)RNA in the affinity-purified His_6_-tagged eEF1Bγ preparation from yeast replicating TBSV repRNA. Both the membrane and soluble yeast fractions were used for eEF1Bγ purification and subsequent RT-PCR analysis to detect (+)repRNA. “+” and “-“ mean that His_6_-tagged eEF1Bγ was expressed from a plasmid or not in yeast. Samples were used for RT-PCR (lanes 3-4 and 7-8) or for PCR (without RT reaction, lanes 1–2 and 5–6).

To test if eEF1Bγ is a component of the tombusvirus replicase, we purified the His_6_-Flag-tagged p33 (HF-p33) replication protein via Flag-affinity purification from the detergent-solubilized membrane fraction of yeast [10]. We detected both p33 and eEF1Bγ in the purified preparation (Figure 5A, lane 1), suggesting that eEF1Bγ is likely part of the replicase complex [21]. Importantly, eEF1Bγ was not found in the control samples containing the His_6_-tagged p33 (H-p33) that were also purified via the Flag-affinity procedure (Figure 5A, lane 2). Since eEF1Bγ does not seem to bind to p33 or p92 replication proteins (data not shown), it is likely that eEF1Bγ was co-purified with p33 via the viral RNA template in the viral replicase complex.

To demonstrate that eEF1Bγ can indeed bind to the TBSV (+)repRNA in cells, we Flag-affinity-purified His_6_-Flag-tagged eEF1Bγ from the detergent-solubilized membrane fraction and also from the soluble (cytosolic) fraction of yeast. Interestingly, the viral RNA was co-purified with eEF1Bγ from both fractions (Figure 5B, lanes 3 and 7). These data confirmed that eEF1Bγ binds to the viral RNA in yeast.

Since eEF1Bγ was found in association with the TBSV repRNA in the cytosolic fraction of yeast, it is possible that eEF1Bγ might affect the viral RNA recruitment from the cytosol into replication that takes place on the peroxisomal or ER membrane surfaces [42], [43]. Therefore, we tested the recruitment of the TBSV (+)repRNA to the membrane fraction in our CFE assay [23]. We found that eEF1Bγ did not facilitate the association of the TBSV (+)repRNA with the membrane when applied in the absence of p33/p92 replication proteins (Figure S2). Moreover, eEF1Bγ did not further increase the amount of TBSV (+)repRNA bound to the membrane in the presence of p33/p92 replication proteins, which are needed for RNA recruitment (Figure S2, lanes 3–4 and 8–10) [24]. Therefore, we conclude that eEF1Bγ is unlikely to promote the recruitment of the TBSV (+)repRNA to the membrane.

### Synergistic effect of eEF1Bγ and eEF1A on the activity of the viral RdRp *in vitro*

**Figure 6:**
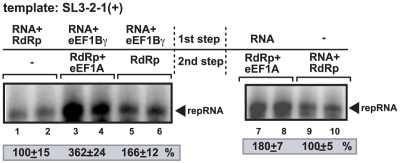
Synergistic effect of eEF1Bγ and eEF1A on stimulation of minus-strand synthesis by the closely-related TCV RdRp. Purified eEF1Bγ (20 pmol) and eEF1A (20 pmol) were added to the TCV RdRp (2 pmol) assay as shown. The RdRp assay had two steps: first, the shown components on the top and bottom were incubated in separate tubes at room temperature to facilitate their interaction, followed 5 min later by mixing the components from the two tubes and addition of the ribonucleotides to start RNA synthesis. The RdRp activity in samples containing the template RNA and the RdRp were chosen as 100% (lanes 1–2 and 9–10). The gel image shows the results of RNA synthesis in the presence of equal amounts of purified eEF1Bγ and eEF1A as shown in a TCV RdRp assay.

Since both eEF1Bγ and eEF1A bind to the 3′-terminal region of the TBSV (+)RNA (Figure 3) and ref: [23], [24], it is possible that they could affect each other's functions during replication. To test the mutual effect of eEF1Bγ and eEF1A on the (-)-strand RNA production of the viral RdRp, we performed *in vitro* RdRp assays with purified eEF1A and recombinant eEF1Bγ as shown in Figure 6. Based on previous experiments, eEF1Bγ was known to stimulate (-)-strand synthesis the most when pre-incubated with the template (+)RNA (Figure 2B). In contrast, pre-incubation of eEF1A with the viral RdRp was more effective than pre-incubation of eEF1A with the template RNA [23]. Therefore, we performed the pre-incubation experiments prior to the RdRp assay as shown in Figure 6. We found the largest stimulation of (-)-strand synthesis by the viral RdRp in a dual pre-incubation assay, when eEF1Bγ was pre-incubated with the viral RNA template, while eEF1A was separately pre-incubated with the viral RdRp (Figure 6, lanes 3–4). Pre-incubation of eEF1Bγ with the viral RNA template (lanes 5–6) or pre-incubation of eEF1A with the viral RdRp (lanes 7–8) were about half as efficient in stimulation of (-)-strand synthesis than the dual pre-incubation assay (lanes 3–4). Therefore, these data support the model that eEF1Bγ and eEF1A both promote (-)-strand synthesis and their effect is synergistic, likely involving separate mechanisms (see Discussion).

### Silencing of eEF1Bγ in plants inhibits TBSV RNA accumulation

**Figure 7:**
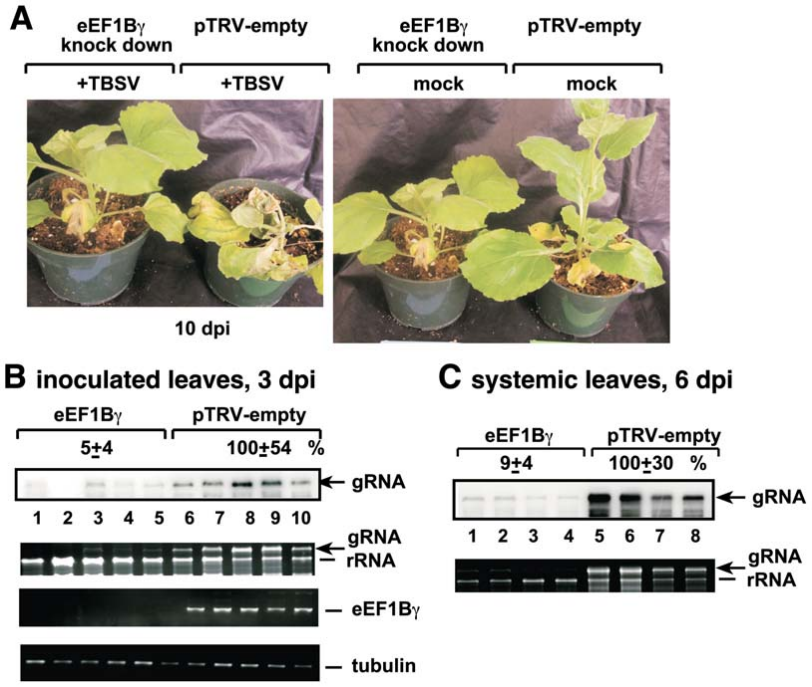
Knockdown of eEF1Bγ inhibits TBSV RNA replication in *N. benthamiana* plants. (A) Symptoms of TBSV infected plants 10 days after inoculation/19 days after agroinfiltration and the phenotype of the eEF1Bγ knockdown *N. benthamiana* plants 19 days after agroinfiltration with the VIGS vectors. VIGS was performed via agroinfiltration of *Tobacco rattle virus* (TRV) vectors carrying eEF1Bγ sequence or the TRV empty vector (as a control). (B) Reduced accumulation of TBSV RNA in the inoculated leaves of eEF1Bγ knockdown *N. benthamiana* plants 3 days post-inoculation, based on Northern blot analysis. Inoculation with TBSV gRNA was done by sap inoculation 9 days after silencing of eEF1Bγ expression. Ribosomal RNA is shown as a loading control at the bottom of the panel. (C) Reduced accumulation of TBSV RNA in the systemically-infected leaves of eEF1Bγ knockdown *N. benthamiana* plants 6 days post-inoculation. See further details in panel B.

To obtain evidence on the importance of eEF1Bγ in TBSV replication in the natural plant hosts, we knocked down the expression of the eEF1Bγ gene in *Nicotiana bethamiana* leaves via VIGS (virus-induced gene silencing). Efficient knocking down of eEF1Bγ mRNA level in *N. benthamiana* (Figure 7B) only resulted in slightly reduced growth of the plants without other phenotypic effects (Figure 7A). The accumulation of TBSV genomic RNA, however, was dramatically reduced in both inoculated (Figure 7B, lanes 1–5) and the systemically-infected young leaves (Figure 7C, lanes 1–4) when compared with the control plants infected with the ‘empty’ *Tobacco rattle virus* (TRV) vector. The lethal necrotic symptoms caused by TBSV in *N. benthamiana* were also greatly attenuated in the eEF1Bγ knock-down plants (Figure 7A). Therefore, we conclude that eEF1Bγ is essential for TBSV genomic RNA accumulation in *N. bethamiana*.

### Silencing of eEF1Bγ in plants inhibits Tobacco mosaic virus RNA accumulation

**Figure 8:**
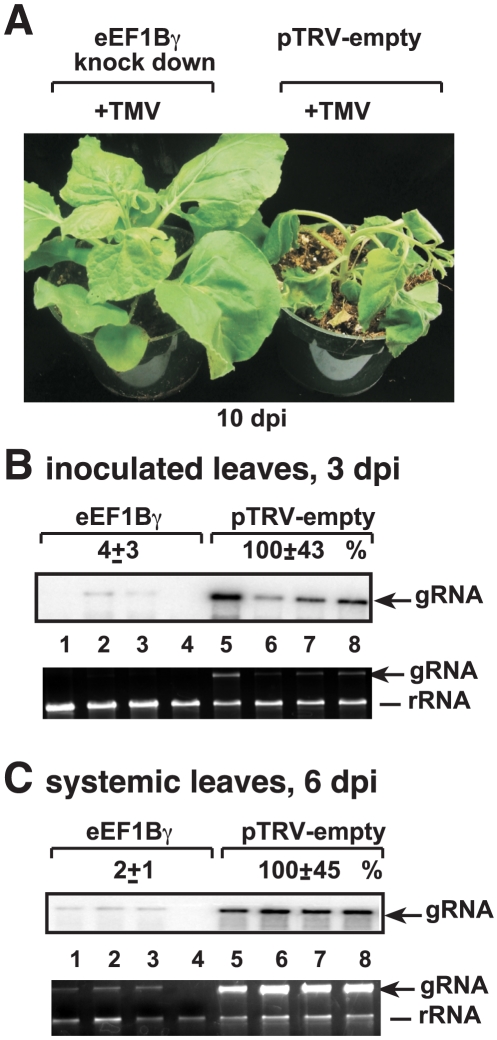
Knockdown of eEF1Bγ inhibits TMV RNA replication in *N. benthamiana* plants. (A) Symptoms of TMV infected plants 10 days after inoculation and 19 days after agroinfiltration with the VIGS vectors. (B) Reduced accumulation of TMV RNA in the inoculated leaves of eEF1Bγ knockdown *N. benthamiana* plants 3 days post-inoculation. (C) Reduced accumulation of TMV RNA in the systemically-infected leaves of eEF1Bγ knockdown *N. benthamiana* plants 6 days post-inoculation. See further details in Figure 7.

To test if eEF1Bγ is also needed for the replication of other plant RNA viruses, we infected eEF1Bγ-silenced *N. benthamiana* leaves with the unrelated *Tobacco mosaic virus* (TMV) RNA (Figure 8A). We found that the severe symptoms caused by TMV were greatly ameliorated in eEF1Bγ knock-down plants (Figure 8A). Accumulation of TMV genomic RNA was also dramatically reduced in both inoculated (Figure 8B) and systemically-infected (Figure 8C) leaves of the eEF1Bγ knock-down plants. Based on these data, eEF1Bγ seems to be needed for TMV replication and/or spread in plants. Thus, our data have revealed new functions for eEF1Bγ in plant RNA virus replication and spread.

## Discussion

Tombusviruses, similar to other (+)RNA viruses, subvert a yet unknown number of host-coded proteins to facilitate robust virus replication in infected cells. The co-opted host proteins could be part of the viral replicase complexes and provide many yet undefined functions. Translation factors, such as eEF1Bγ and eEF1A, are among the most common host factors recruited for (+)RNA virus replication [23], [24]. While eEF1A is an integral component of the tombusvirus replicase complex [23], [24] and several other viral replicases [44], [45], [46], the function of eEF1Bγ in tombusvirus replication is studied in this paper. Co-purification experiments with the p33 replication protein, which is the most abundant protein component in the tombusvirus replicase complex [21], [22], revealed that eEF1Bγ is a permanent member of the replicase (Figure 5A). eEF1Bγ is likely recruited into the viral replicase via the viral (+)RNA, which is bound to eEF1Bγ in both cytosolic and membranous fractions (Figure 5B). The possible role of host proteins or membrane lipids in assisting the recruitment of eEF1Bγ for TBSV replication cannot be excluded. Accordingly, eEF1Bγ has been shown to bind to a large number of host proteins (www.yeastgenome.org). For example, eEF1A, which is also a permanent member of the tombusvirus replicase, is known to interact with eEF1Bγ [47], [48], [49] and eEF1A might facilitate the recruitment of eEF1Bγ and possibly other translation factors. The binding of eEF1Bγ to intracellular membranes has also been shown before [32]. Altogether, our model predicts that the viral (+)RNA could be involved in recruitment of eEF1Bγ into viral replication (Figure 5). However, the opposite model that eEF1Bγ facilitates the recruitment of the TBSV (+)RNA into replication is not supported by our *in vitro* data (Figure S2). Indeed, addition of eEF1Bγ to the CFE assay did not increase the membrane-bound fraction of TBSV (+)repRNA in the absence or presence of the viral replication proteins (Figure S2).

### eEF1Bγ selectively enhances minus-strand synthesis by opening the closed 3′-terminus during TBSV RNA replication

We confirmed a direct role for eEF1Bγ in RNA synthesis *in vitro* by using a cell-free extract prepared from *tef4*Δ yeast that supported (-)-strand RNA synthesis ∼3-fold less efficiently than CFE from wt yeast (Figure 1). Moreover, *in vitro* assays with highly purified eEF1Bγ and the recombinant TCV RdRp, which is closely homologous with the TBSV p92^pol^, also revealed that eEF1Bγ stimulates (-)-strand synthesis by binding to the viral (+)RNA template (Figure 3). Accordingly, pre-incubation of eEF1Bγ and the TBSV-derived template RNA prior to the RdRp assay led to the highest level of stimulation of (-)RNA synthesis (Figure 2). On the other hand, eEF1Bγ does not stimulate the RdRp activity directly, since pre-incubation of eEF1Bγ with the RdRp did not lead to more efficient (-)-strand RNA synthesis *in vitro* (Figure 2). We propose that eEF1Bγ modifies the structure of the (+)-strand template prior to initiation of (-)-strand synthesis that leads to more efficient RNA synthesis as described below.

**Figure 9:**
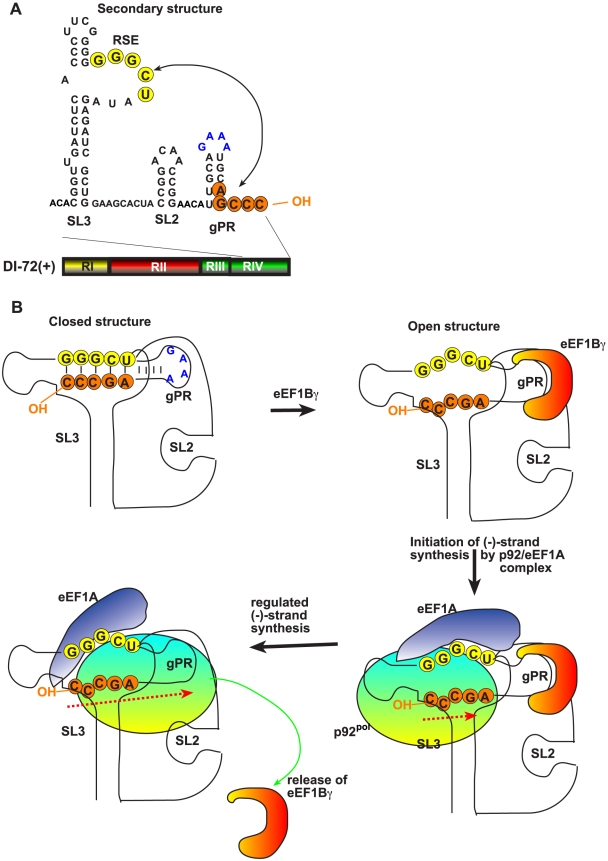
A model describing the functions of eEF1Bγ and eEF1A during tombusvirus replication. (A) Schematic representation of the secondary structure of the TBSV 3′ end and the RSE-gPR interaction. The arrow indicates the middle-range RNA base-pairing that leads to a closed structure formed at the 3′ end of TBSV RNA as shown in panel B. (B) We propose that eEF1Bγ opens up the closed structure in the RNA, while eEF1A binds to both the p92^pol^ replication protein and the RSE sequence. These events lead to proper positioning of p92^pol^ over the “opened” promoter sequence, thus facilitating initiation of minus-strand synthesis. Displacement of eEF1Bγ from the (+)RNA during RNA synthesis decreases the probability of new rounds of initiation by p92^pol^ on the original (+)RNA templates. Thus, these events favor limited minus-strand synthesis and facilitate plus-strand synthesis to generate excess amount of new (+)-strands.

*In vitro* initiation of (-)-strand synthesis by the viral RdRp requires the gPR promoter consisting of a short 3′-terminal single-stranded tail and a stem-loop (SL1) sequence [39], [50]. However, the gPR region is present in a ‘closed’ structure in the TBSV (+)RNA due to base-pairing of a portion of the gPR with the RSE present in SL3 as shown in Figure 9. This interaction makes the TBSV (+)RNA poor template in the *in vitro* assay due to the difficulty for the viral RdRp to recognize and/or open the ‘closed’ structure [39]. Our current work with eEF1Bγ, however, suggests that eEF1Bγ can bind to the tetraloop region of SL1 (and to an A-rich sequence in SL2) that leads to melting of the base-paired structure and opening the stem of SL1 and the RSE-gPR base-pairing as shown schematically in Figure 9B. We propose that the open structure can be recognized efficiently by the viral replicase leading to efficient initiation of (-)-strand synthesis (Figure 9B). This model is supported by several pieces of evidence presented in this paper, including (i) stimulation of (-)-strand synthesis by eEF1Bγ when the wt SL1 is present in the template; (ii) lack of stimulation of(-)-strand synthesis by eEF1Bγ when a mutated SL1 (tetraloop mutant), which does not bind efficiently to eEF1Bγ, was used as a template in the *in vitro* assay; (iii) stimulation of (-)-strand synthesis when eEF1Bγ was pre-incubated with the (+)-strand template, but not when eEF1Bγ was pre-incubated with the viral RdRp (Figure 2); and (iv) the lack of stimulation of (+)-strand synthesis on a (-)-strand template by eEF1Bγ (Figure 2). In addition, eEF1Bγ stimulated (-)-strand synthesis by the viral RdRp when a partially complementary RNA oligo was hybridized with the SL1 region (Figure 4B). However, eEF1Bγ could not efficiently bind to the 3′-end of the TBSV RNA when it formed a hybrid (duplex) with a perfectly complementary DNA oligo (Figure 3A), suggesting that eEF1Bγ can melt only the local secondary structure, but cannot unwind more extended duplex regions. An alternative possibility is that eEF1Bγ protein stabilizes the unpaired structure (when the SL1 structure is kinetically pairing/unpairing), rather than implying that it actively "opens" the structure.

An intriguing aspect of our model is the possible regulation of the “open” and “closed” structure of the 3′ UTR by eEF1Bγ. Displacement of eEF1Bγ bound to the 3′-end by the viral replicase during (-)-strand synthesis could make the 3′-terminus of the (+)-strand RNA fold back into a ‘closed’ structure. This could prevent efficient re-utilization of the original (+)-strand template during TBSV replication, and the switch to efficient (+)-strand synthesis on the (-)RNA intermediate (Figure 9B). This model can also explain why the newly made (+)-strand RNA progeny will not enter the replication cycle in the absence of bound eEF1Bγ within the originally-formed replicase complexes as observed previously in the CFE assay [20]. We propose that the new (+)RNA progeny need to leave the replicase complex, then bind to eEF1Bγ in the cytosol and assemble new replicase complexes, followed by a new round of viral RNA replication. Thus, this model suggests that eEF1Bγ plays a key role in regulation of the use of (+)-strand RNAs in TBSV replication (Figure 9B).

Our finding of TBSV RNA binding by eEF1Bγ adds to the growing list of RNAs bound by eEF1Bγ. For example, the 3′ UTR of vimentin mRNA is bound by eEF1Bγ [51], which led the authors to suggest that eEF1Bγ plays a role in vimentin mRNA subcellular localization by also binding to cytoskeleton or membranes. eEF1Bγ also binds to the tRNA-like structure at the 3′ UTR of BMV, albeit the relevance of this binding is currently unclear [51]. Also, the actual role of eEF1Bγ in the VSV replicase is currently not defined [31].

Translation elongation factors seem to be important for replication of many RNA viruses. For example, EF-Tu and EF-Ts play a role in replication of bacteriophage Qbeta [52], [53]. The eukaryotic homolog of EF-Tu, eEF1A was found to bind to viral RNAs, such as TBSV, *Turnip yellow mosaic virus* (TYMV) [54], West Nile virus (WNV), Dengue virus, hepatitis delta virus, TMV, *Brome mosaic virus*, and *Turnip mosaic virus* [55], [56], [57], [58], [59], [60] and to viroid RNAs [61]. Therefore, it is highly probable that many (+)-strand RNA viruses recruit translation elongation factors to facilitate and regulate their replication in infected cells.

### Nonoverlapping roles of eEF1Bγ and eEF1A in stimulation of (-)-strand synthesis

The emerging picture on the functions of eEF1Bγ and eEF1A is that these translation elongation factors play different, yet complementary roles in TBSV replication as suggested in Figure 9B. While eEF1Bγ binds to SL1, eEF1A has been shown to bind to both p92^pol^ RdRp and the SL3 region of TBSV (+)repRNA [23], [24]. The binding of the RNA by eEF1Bγ promotes the opening of the closed 3′-terminal structure, whereas eEF1A facilitates the proper and efficient binding of the RdRp to the 3′ terminal RSE sequence of the viral RNA, which is required for the assembly of the viral replicase complex [11], [39], prior to initiation of (-)-strand synthesis (Figure 9) [23], [24]. The binding of eEF1A-RdRp complex to the RSE might lead to proper positioning of the RdRp over the 3′-terminal gPR promoter sequence opened up by eEF1Bγ, thus facilitating the initiation of (-)RNA synthesis starting from the 3′-terminal cytosine (Figure 9B). Altogether, the two translation factors facilitate the efficient initiation of (-)-strand synthesis in addition to reducing the possibility of re-utilization of the (+)-strand template for additional rounds of (-)-strand synthesis. This regulation of RNA synthesis by the co-opted host factors shows the specialized use of host components to serve the need of viral replication.

### eEF1Bγ is required for TBSV replication in yeast and plant hosts

The current work also provides evidence that eEF1Bγ is a key factor in TBSV replication in yeast (Figure 1) and in *N. benthamiana* (Figure 7). Since eEF1Bγ is a highly conserved protein in all eukaryotes [32], it is not surprising that yeast eEF1Bγ, similar to the plant eEF1Bγ, can be co-opted for TBSV replication. Interestingly, deletion of either *TEF3* or *TEF4* genes reduced TBSV repRNA accumulation in yeast, suggesting that eEF1Bγ is present in limiting amount or eEF1Bγ is present in not easily accessible forms (in protein complexes) and/or locations in yeast cells. Silencing of eEF1Bγ in *N. bethamiana* showed even more inhibition of TBSV RNA accumulation than deletion of eEF1Bγ genes in yeast. This is likely due to the robust antiviral response (i.e., induced gene silencing) of the plant host, which could result in degradation of the small amount of viral RNA produced by the less efficient viral RNA replication in the presence of limited eEF1Bγ in the knock-down plants.

Silencing of eEF1Bγ expression in *N. benthamiana* also reduced the accumulation of the unrelated TMV (Figure 8), which belongs to the alphavirus-like supergroup. These data suggest that eEF1Bγ is likely involved in TMV replication, which also contains a highly structured 3′- end [54]. Therefore, it is possible that eEF1Bγ is co-opted by different plant RNA viruses, and possibly other RNA viruses as well.

### Conclusion

Overall, the current work suggests three major functions for eEF1Bγ in TBSV replication (Figure 9): (i) enhancement of the minus-strand synthesis by opening the ‘closed’ 3′-end of the template RNA; (ii) reducing the possibility of re-utilization of (+)-strand templates for repeated (-)-strand synthesis; and (iii) in coordination with eEF1A, stimulation of the proper positioning of the viral RdRp over the promoter region in the viral RNA template. These roles for eEF1Bγ and eEF1A are separate from their canonical roles in host and viral protein translation.

## Materials and Methods

### Yeast strains and expression plasmids

*Saccharomyces cerevisiae* strain BY4741 (*MAT*a *his3Δ1 leu2Δ0 met15Δ0 ura3Δ0*) and the single-gene deletion strain of the *TEF4*-encoded form of eEF1Bγ (*tef4*Δ) were obtained from Open Biosystems (Huntville, AL). TKY680 strain in which both yeast encoded eEF1Bγ, *TEF4* and *TEF3* were deleted (*MAT*a *ura3-52 leu2Δ1 his3Δ200 trp1Δ101 lys2-801 tef3::LEU2 tef4::TRP1*) and its isogenic wild type TKY677 (*MAT*a *ura3-52 leu2Δ1 his3Δ200 trp1Δ101 lys2-801*) as well as the isogenic single deletion mutant strains, TKY678 (*MAT*a *ura3-52 leu2Δ1 his3Δ200 trp1Δ101 lys2-801 tef3::LEU2*) and TKY 679 (*MAT*a *ura3-52 leu2Δ1 his3Δ200 trp1Δ101 lys2-801 tef4::TRP1)* were published previously [30]. The following plasmids pESC-GAL1-Hisp33/GAL10-DI-72, pGAD-CUP1-p92 pYES-GAL1-p92, pCM189-TET-His92 were described earlier [21], [22]. *URA3* based pGBK-ADH- Hisp33/GAL1-DI72, pGBK-CUP1-HisFLAGp33/GAL1-DI-72, and pGBK-CUP1- Hisp33/GAL1-DI-72 plasmids were constructed by Daniel Barajas (unpublished result). The *URA3* based, low copy-number plasmid, pYC-GAL1-Tef4 expressing non-tagged full-length Tef4 protein was constructed as follows: pYC/NT-C plasmid was digested with *BamH*I and *Xho*I restriction enzymes and then PCR product of the *TEF4* gene was generated with primers #2089 (ccgcGGATCCATGTCCCAAGGTACTTTATAC) and #2320 (CGCCTCGAGTTATTTCAAAACCTTACCGTCAACAATTTCC) and digested with the same restriction enzymes, followed by ligation. The plasmid pYES-NTC2-GAL1-HisTef4 expressing His_6_-tagged Tef4p protein was created with the same restriction enzymes using pYES-NT-C2.

*HIS3*-based pEsc-His/Cup-FLAG plasmid [20] was digested with *BamH*I and *Xho*I restriction enzymes and then PCR product of the *TEF4* gene was generated with primers #2089 and #2320 and digested with the same restriction enzymes, followed by ligationto obtain pEsc-His/Cup-FLAG-TEF4.

### *In vivo* replication assay

*HIS3* based pESC-GAL1-His33/GAL10-DI-72 and *LEU2* based pGAD-CUP1-Hisp92 plasmids were transformed into *tef4*Δ strain. In the *in vivo* complementation assay, non-tagged Tef4p protein was expressed from *URA3* plasmid pYC-GAL1-Tef4 and *TEF4* mRNA was detected with a specific probe generated by the T7 transcription of the PCR product obtained with primers #2089 and #3788 (TAATACGACTCACTATAGGATTATTTCAAAACCTTACCGTCAACAATTTCC).

TKY680 (*tef3Δ/tef4Δ*), the isogenic TKY679 (*tef4Δ*), TKY678 (*tef3Δ*) and wild type TKY677 yeast were transformed with plasmids pESC-GAL1-His33/GAL10-DI-72 and pCM189-TET-His92. Yeast was pre-grown at 23°C overnight in 3 ml synthetic complete dropout medium lacking the relevant amino acids containing 2% glucose and 1 mg/ml doxycyclin to suppress p92 expression by the inhibition of TET promoter and then TBSV replication was launched by replacing the media with 2% galactose without doxycycline. Cells were harvested at 48 h time point. Total RNA extraction from yeast cells and Northern blotting and Western blotting were done as previously described [15], [24].

### Expression and purification of recombinant eEF1Bγ protein

pEsc-His/Cup-FLAG-TEF4 plasmid was transformed into *tef4*Δ strain. Yeast was pre-grown overnight at 29°C in 2 ml synthetic complete dropout medium lacking histidine (SC-H^-^ medium) containing 2% glucose. The volume of the media was increased up to 100 ml 16 h later and copper sulfate was added to a final concentration of 50 µM for induction of protein expression. Yeast was grown to 0.8 OD_600_ (∼4–6 h). Then, yeast cells were harvested and broken by glass beads in a FastPrep cell disruptor followed by Flag-affinity purification of FLAG-Tef4p protein [34]. The bacterial heterologous expression and purification of His_6_-tagged Tef3 protein from plasmid pTKB523 was performed as described in ref: [62] using only the Ni affinity column step.

### Tombusvirus replication assay using yeast cell free extract

Yeast extract capable of supporting TBSV replication *in vitro* was prepared as described [20]. The newly synthesized ^32^P-labeled RNA products were separated by electrophoresis in a 5% polyacrylamide gel (PAGE) containing 0.5x Tris-borate-EDTA (TBE) buffer with 8 M urea. To detect the double-stranded RNA (dsRNA) in the cell-free replication assay, the ^32^P-labeled RNA samples were divided into two aliquotes: one half was loaded onto the gel without heat treatment in the presence of 25% formamide, while the other half was heat denatured at 85°C for 5 min in the presence of 50% formamide [20].

To test the *in vitro* activity of Tef4p, different concentrations (26 and 13 pmol) of purified FLAG/His_6_-Tef4p was added to 0.25 µg (4 pmol) DI-72 (+)repRNA transcript and incubated in the presence of yeast cell-free extract and reaction buffer for 10 minutes at RT followed by the addition of MBP-p33 and MBP-p92 along with the rest of the reaction components. The reaction was performed at 25°C for 3 h and analyzed as above.

### *In vitro* TCV p88C RdRp assay

The TCV RdRp reactions were carried out as previously described for 2 h at 25°C [36], except using 7 pmol template RNA and 2 pmol affinity-purified MBP-p88C. Different concentrations of eEF1Bγ (6xHis-affinity purified recombinant Tef3p obtained from *E. coli or* Flag-affinity purified HF-Tef4p obtained from yeast*)* were added to the reaction at the beginning or as indicated in the text and Figure 2. legend. The ^32^P-labeled RNA products were analyzed by electrophoresis in a 5% PAGE/8 M urea gel [63]. The 86-nt 3′ noncoding region of TBSV genomic RNA and its mutants were used as the template in the RdRp assay [24], [36]. RNA templates were generated with T7 transcription using PCR products obtained with the following primers: #1662 (TAATACGACTCACTATAGGACACGGTTGATCTCACCCTTC) and #1190 (GGGCTGCATTTCTGCAATG) for SL3-2-1(+), #1662 and #4390 (GGGCTGCACAAGTGCAATGTTCCGGTTGTCCGGT) for SL3-2-1cuug(+). SL3-2-1m(+) RNA was generated with T7 transcription on PCR products amplified with primers #1662 and #1190, on a plasmid template harboring GGGCU nucleotide-deletion in SL3 region as described [39]. A duplex RNA was generated by hybridizing SL3-2-1(+) and SL3-2-ds1(-) made by T7 transcription of the PCR product using primers #4361 (GTAATACGACTCACTATAGGGCTACTTCCGGTTGTCCGGTAGTGCTTCC) and # 4362 (CGGTTGATCTGACCCTTCGG). For hybridization, equal amounts of both RNAs were mixed in 1X STE buffer [0.1 M NaCl 10 mM Tris-HCl (pH 8.0) 1 mM EDTA (pH 8.0)] followed by treatments: 94°C for 15 s, 70 cycles with gradually lowering the temperature by 1°C at each cycle for 30 s and finally 20°C for 30s.

### Gel mobility shift assay (EMSA) with eEF1Bγ

For EMSA, 6xHis-Flag tagged Tef4p was purified from a yeast *tef4*Δ strain with anti-FLAG M2-agarose affinity resin. Different concentrations (0.6, 0.5 and 0.4 pmol) of HF-Tef4p protein was used for incubation with 0.2 pmol of ^32^P-labeled SL3/2/1(+) RNA or mutated RNAs at 25°C in a binding buffer [50 mM Tris-HCl (pH 8.2), 10 mM MgCl_2_, 10 mM DTT, 10% glycerol, 2 U of RNase inhibitor (Ambion)]. Samples were incubated at 25°C for 15 min, then resolved in 4% nondenaturing polyacrylamide gel [23]. Similar experiments were also performed with 6xHis-affinity purified recombinant Tef3p obtained from *E. coli* (not shown).

### Flag-affinity purification of eEF1Bγ-TBSV repRNA complex

For the co-purification of TBSV DI-72 repRNA and eEF1Bγ protein, the yeast *tef4*Δ strain was co-transformed with pGBK-ADH-Hisp33/GAL1-DI72, pGAD-CUP1-Hisp92 and pESC-CUP1-HisFLAG-Tef4. The pESC-CUP1-FLAGHis-Tef4 plasmid was replaced with the pESC plasmid in the control experiment. Yeast was pre-grown overnight at 29°C in 2 ml SC^ULH-^ medium containing 2% glucose and 5 µM copper sulfate. The volume of the media was increased to 20 ml after 16 h for an additional 10 h (OD_600_ of ∼0.8), then the cultures were transferred to 20 ml SC^ULH-^ medium containing 2% galactose to induce TBSV DI-72 RNA transcription at 23°C. The transcription of DI-72 RNA was stopped by changing to the media containing 2% glucose after 8 h. The cultures were diluted to 200 ml and copper sulfate was added to a final concentration of 50 µM to induce the expression of Flag-tagged Tef4 protein. After incubation at 23°C for 24 h, the samples were centrifuged at 3000 rpm for 4 min. Cells (∼1 g) were re-suspended in 2 ml TG Buffer (50 mM Tris–HCl [pH 7.5], 10% glycerol, 15 mM MgCl_2_, and 10 mM KCl) supplemented with 0.5 M NaCl and 1% [V/V] YPIC yeast protease inhibitor cocktail (Sigma) and RNase inhibitor (Ambion). Yeast cells were broken by glass beads in a FastPrep cell disruptor (MP Biomedicals) 4 times for 20 sec each at speed 5.5. Samples were removed and incubated 1 min in an ice-water bath after each treatment. The samples were centrifuged at 500 ×g for 5 min at 4°C to remove glass beads, unbroken cells and debris then supernatant was moved into fresh pre-chilled tubes. After being centrifuged again at 500 ×g for 5 min at 4°C supernatant transferred into fresh pre-chilled tubes and soluble (SU) and membrane (ME) fractions containing the viral replicase complex were separated with centrifugation at 35,000 ×g for 15 min at 4°C. The SU fraction was applied on 0.1 ml anti-FLAG M2-agarose affinity resin (Sigma) and Tef4 protein tagged with 6xHis- and FLAG affinity tags was purified. Before applying ME fraction on the anti-FLAG M2 resin, solubilization of the membrane-bound replicase was performed in 1 ml TG buffer with 0.5 M NaCl, 1% [V/V] YPIC yeast protease inhibitor cocktail (Sigma), and 2% Triton X-100 via rotation for 2 hours at 4 °C. The solubilized membrane fraction was centrifuged at 35,000 ×g at 4°C for 15 min and the supernatant was added to the resin pre-equilibrated with TG buffer supplemented with 0.5 M NaCl and 0.5% Triton X-100, followed by gentle rotation for 2 h at 4°C. The unbound proteins were removed by gravity flow, and the resin was washed two times with 1 ml TG buffer supplemented with 0.5 M NaCl, 0.5% Triton X-100 and once with 1 ml TG buffer, 0.5% Triton without NaCl. The bound proteins were eluted with 150 µl TG buffer without NaCl, 0.5% Triton X-100, supplemented with 150 µg/ml flag peptide and 1% yeast protease inhibitor cocktail via gentle tapping the column occasionally for 2 h at 4°C. After centrifugation at 600 ×g 2 min at 4°C, semi-quantitative RT-PCR was performed to detect TBSV repRNA co-purified with eEF1Bγ using primers, #359 (GTAATACGACTCACTATAGGAAATTCTCCAGGATTTC) and #1190, amplifying full length (+)repRNA.

### Purification of the viral replicase

To test if eEF1Bγ is present in the viral replicase, yeast *tef4*Δ strain was transformed with pGBK-CUP1-HisFLAGp33/GAL1-DI-72, pGAD-CUP1-Hisp92 and pYES-GAL1-HisTef4. In the control experiment, 6xHisp33was expressed from pGBK-CUP1-Hisp33/GAL1-DI-72. Yeast cultures were grown in SC-ULH^-^ media containing 1% raffinose and 1% galactose with 5 µM copper-sulfate for 4 days with increasing the volume of the culture from 2 ml to 100 ml to a final OD_600_ of∼ 1.0. After harvesting of cells, co-purification of 6xHis-tagged Tef4p with HF-p33 (part of the viral replicase) was conducted by using anti-FLAG M2-agarose affinity resin as described above (in the section: FLAG-affinity purification of eEF1Bγ-TBSV repRNA complex), with the exception that only solubilized ME fraction was loaded on the column. Proteins bound to affinity resin were eluted by incubation with 150 µl buffer containing FLAG peptide and precipitated with Trichloroacetic acid (TCA) [64]. Samples were analyzed by SDS-PAGE and Western blotting.

### Virus induced gene silencing of eEF1Bγ in *N. benthamiana* plants

Virus-induced gene silencing (VIGS) in *N. benthamiana* was done as described [65], [66]. To generate the VIGS vector (pTRV2- eEF1BγNt), a 314-bp cDNA fragment of NteEF1Bγ was RT-PCR amplified from a total RNA extract of *N. benthamiana* using the following pair of primers: #2993 (CGCGGATCCAAAGGTTTCTGGGACATGTATGA) and #2994 (CGCCTCGAGACACGCTCCTTCTGTGATTCATC) and inserted into the corresponding (*BamH*I/*Xho*I) restriction sites of pTRV2 plasmid.

The sequence of the *N. tabacum* eEF1Bγ gene (GenBank: ACB72462.1) was derived via a BLASTP search based on the C- terminal (translation elongation factor) domain (aa 252–412) of the *Saccharomyces cerevisie* Tef4 protein. The selected sequence (TC64920) from the Solanaceae Genomics Resource (www.tigr.org) gave 98% identity with *N. tabacum* EF1Bγ -like gene (GB#: EU580435.1).

To confirm the silencing of the EF1Bγ gene in *N. benthamiana*, we performed RT-PCR amplification with primer pairs: #2952 (CGCGGATCCGGAAAGGTTCCTGTGCTTGA) and #2992 (CGCCTCGAGGTCCAGAAGTATCTCTCTACATGTGG) on total RNA extract of pTRV2- EF1BγNt and pTRV2_empty_ agro-infiltrated *N benthamiana* plants. PCR conditions were as follows: 27 cycles of 94°C 20sec, 60°C 30sec, 68°C 30 sec with HiFi Taq polymerase. Tubulin mRNA control from the same total RNA samples was detected by RT-PCR using primers #2859 (TAATACGACTCACTATAGgaACCAAATCATTCATGTTGCTCTC) and #2860 (TAGTGTATGTGATATCCCACCAA) [65]. The leaves of VIGS-treated plants were sap inoculated with TBSV, or TMV on the 9^th^ day after silencing [65]. Total RNA was extracted 3 or 5 days post inoculation [65]. For Northern blot analysis of the viral RNA level, we prepared ^32^P-labeled complementary RNA probes specific for the 3′-ends of the viral genomic RNAs based on T7 transcription. To obtain the PCR templates for the probes, we used the following primers for TBSV: #1165 (AGCGAGTAAGACAGACTCTTCA) and #22; for TMV: #2890 (TCTGGTTTGGTTTGGACCTC) and #2889 (GTAATACGACTCACTATAGGGATTCGAACCCCTCGCTTTAT).

### *In vitro* viral RNA recruitment assay

The TBSV viral RNA is recruited to the membrane from the soluble fraction with the help of TBSV replication proteins and host factors present in the yeast CFE. The *in vitro* RNA recruitment reaction was performed according to [20], [23], except that ^32^P-labeled DI-72 (+)repRNA were used and rCTP, rUTP, ^32^P-labeled UTP, and Actinomycin D were omitted from the assay. As a negative control, p33 and p92 were omitted from the reaction to detect DI-72 binding nonselectively to host proteins present in the membrane.

## Supporting Information

Figure S1 eEF1Bγbinds to the 3′ end of the TBSV (+)RNA. (A) *In vitro* binding assay with purified recombinant eEF1Bγ (Tef3). The TBSV (+)RNA templates were the four noncontiguous segments of the TBSV (+)RNA that are present in defective interfering RNAs, including DI-72 repRNA used in this study. RI(+) represents the 5′-UTR, RII(+)-SL is an internal highly conserved sequence that binds to p33 replication protein, RIII(+) is ashort conserved sequence closed to the 3′ end, andSL3-2-1(+), which contains the promoter region (SL1) for initiation and the replication silencer element (within SL3) that down-regulates initiation. The assay contained ^32^P-labeled free ssRNA (as shown), plus 0.6 pmol purified recombinant eEF1Bγ, respectively. The bound RNA-protein complexes were separated on nondenaturing 5% acrylamide gels. Quantification of the free (unshifted) RNA was done with ImageQuant. (B) RNA gel shift analysis shows SL3-2-1(+) RNA binds competitively to eEF1Bγ. The RNA templates representing the 3′ end of the TBSV RNA and the deleted nucleotides are shown schematically. The cold competitor was SL3-2-1(+) RNA, which represents a large portion of the 3′-UTR (Figure 4A). The eEF1Bγ - ^32^P-labeled ssRNA complex was visualized on nondenaturing 5% acrylamide gels.
(EPS)

Figure S2 eEF1Bγdoes not affect the template recruitment step *in vitro*. Purified recombinant p33/p92 and ^32^P-labeled DI-72 (+)repRNA and eEF1Bγ (affinity purified recombinant Tef3) were added to a whole cell extract (CFE), followed by centrifugation/washing to remove the ^32^P-labeled repRNA that is not bound to the membrane. Then the membrane-bound RNA was analyzed in a denaturing PAGE gel. Note that the repRNA binds to the cellular membrane fraction nonspecifically (∼20% level) in the absence of the viral replication proteins.
(EPS)

## References

[1] Noueiry AO, Ahlquist P (2003). Brome Mosaic Virus RNA Replication: Revealing the Role of the Host in RNA Virus Replication.. Annu Rev Phytopathol.

[2] Nagy PD (2008). Yeast as a model host to explore plant virus-host interactions.. Annu Rev Phytopathol.

[3] Strauss JH, Strauss EG (1999). Viral RNA replication. With a little help from the host.. Science.

[4] Wang RY, Nagy PD (2008). Tomato bushy stunt virus Co-Opts the RNA-Binding Function of a Host Metabolic Enzyme for Viral Genomic RNA Synthesis.. Cell Host Microbe.

[5] Li Z, Nagy PD (2011). Diverse roles of host RNA binding proteins in RNA virus replication.. RNA Biol.

[6] Nagy PD, Pogany J (2006). Yeast as a model host to dissect functions of viral and host factors in tombusvirus replication.. Virology.

[7] Brinton MA (2001). Host factors involved in West Nile virus replication.. Ann N Y Acad Sci.

[8] Shi ST, Lai MM (2005). Viral and cellular proteins involved in coronavirus replication.. Curr Top Microbiol Immunol.

[9] Panavas T, Nagy PD (2003). Yeast as a model host to study replication and recombination of defective interfering RNA of Tomato bushy stunt virus.. Virology.

[10] Panaviene Z, Panavas T, Serva S, Nagy PD (2004). Purification of the cucumber necrosis virus replicase from yeast cells: role of coexpressed viral RNA in stimulation of replicase activity.. J Virol.

[11] Panaviene Z, Panavas T, Nagy PD (2005). Role of an internal and two 3′-terminal RNA elements in assembly of tombusvirus replicase.. J Virol.

[12] Pogany J, White KA, Nagy PD (2005). Specific Binding of Tombusvirus Replication Protein p33 to an Internal Replication Element in the Viral RNA Is Essential for Replication.. J Virol.

[13] Nagy PD, Pogany J (2008). Multiple roles of viral replication proteins in plant RNA virus replication.. Methods Mol Biol.

[14] Kushner DB, Lindenbach BD, Grdzelishvili VZ, Noueiry AO, Paul SM (2003). Systematic, genome-wide identification of host genes affecting replication of a positive-strand RNA virus.. Proc Natl Acad Sci U S A.

[15] Panavas T, Serviene E, Brasher J, Nagy PD (2005). Yeast genome-wide screen reveals dissimilar sets of host genes affecting replication of RNA viruses.. Proc Natl Acad Sci U S A.

[16] Jiang Y, Serviene E, Gal J, Panavas T, Nagy PD (2006). Identification of essential host factors affecting tombusvirus RNA replication based on the yeast Tet promoters Hughes Collection.. J Virol.

[17] Nagy PD, Pogany J (2010). Global genomics and proteomics approaches to identify host factors as targets to induce resistance against tomato bushy stunt virus.. Adv Virus Res.

[18] Wang RY, Stork J, Pogany J, Nagy PD (2009). A temperature sensitive mutant of heat shock protein 70 reveals an essential role during the early steps of tombusvirus replication.. Virology.

[19] Wang RY, Stork J, Nagy PD (2009). A key role for heat shock protein 70 in the localization and insertion of tombusvirus replication proteins to intracellular membranes.. J Virol.

[20] Pogany J, Stork J, Li Z, Nagy PD (2008). In vitro assembly of the Tomato bushy stunt virus replicase requires the host Heat shock protein 70.. Proc Natl Acad Sci U S A.

[21] Serva S, Nagy PD (2006). Proteomics analysis of the tombusvirus replicase: Hsp70 molecular chaperone is associated with the replicase and enhances viral RNA replication.. J Virol.

[22] Li Z, Barajas D, Panavas T, Herbst DA, Nagy PD (2008). Cdc34p Ubiquitin-Conjugating Enzyme Is a Component of the Tombusvirus Replicase Complex and Ubiquitinates p33 Replication Protein.. J Virol.

[23] Li Z, Pogany J, Tupman S, Esposito AM, Kinzy TG (2010). Translation Elongation Factor 1A Facilitates the Assembly of the Tombusvirus Replicase and Stimulates Minus-Strand Synthesis.. PLoS Pathog.

[24] Li Z, Pogany J, Panavas T, Xu K, Esposito AM (2009). Translation elongation factor 1A is a component of the tombusvirus replicase complex and affects the stability of the p33 replication co-factor.. Virology.

[25] Pathak KB, Sasvari Z, Nagy PD (2008). The host Pex19p plays a role in peroxisomal localization of tombusvirus replication proteins.. Virology.

[26] Barajas D, Nagy PD (2010). Ubiquitination of tombusvirus p33 replication protein plays a role in virus replication and binding to the host Vps23p ESCRT protein.. Virology.

[27] Barajas D, Jiang Y, Nagy PD (2009). A Unique Role for the Host ESCRT Proteins in Replication of Tomato bushy stunt virus.. PLoS Pathog.

[28] Mateyak MK, Kinzy TG (2010). eEF1A: thinking outside the ribosome.. J Biol Chem.

[29] Esposito AM, Kinzy TG (2010). The eukaryotic translation elongation Factor 1Bgamma has a non-guanine nucleotide exchange factor role in protein metabolism.. J Biol Chem.

[30] Olarewaju O, Ortiz PA, Chowdhury WQ, Chatterjee I, Kinzy TG (2004). The translation elongation factor eEF1B plays a role in the oxidative stress response pathway.. RNA Biol.

[31] Das T, Mathur M, Gupta AK, Janssen GM, Banerjee AK (1998). RNA polymerase of vesicular stomatitis virus specifically associates with translation elongation factor-1 alphabetagamma for its activity.. Proc Natl Acad Sci U S A.

[32] Le Sourd F, Boulben S, Le Bouffant R, Cormier P, Morales J (2006). eEF1B: At the dawn of the 21st century.. Biochim Biophys Acta.

[33] Kinzy TG, Ripmaster TL, Woolford JL (1994). Multiple genes encode the translation elongation factor EF-1 gamma in Saccharomyces cerevisiae.. Nucleic Acids Res.

[34] Pogany J, Nagy PD (2008). Authentic replication and recombination of Tomato bushy stunt virus RNA in a cell-free extract from yeast.. J Virol.

[35] Cheng CP, Panavas T, Luo G, Nagy PD (2005). Heterologous RNA replication enhancer stimulates in vitro RNA synthesis and template-switching by the carmovirus, but not by the tombusvirus, RNA-dependent RNA polymerase: implication for modular evolution of RNA viruses.. Virology.

[36] Rajendran KS, Pogany J, Nagy PD (2002). Comparison of turnip crinkle virus RNA-dependent RNA polymerase preparations expressed in Escherichia coli or derived from infected plants.. J Virol.

[37] Cheng CP, Nagy PD (2003). Mechanism of RNA recombination in carmo- and tombusviruses: evidence for template switching by the RNA-dependent RNA polymerase in vitro.. J Virol.

[38] Nagy PD, Pogany J (2000). Partial purification and characterization of Cucumber necrosis virus and Tomato bushy stunt virus RNA-dependent RNA polymerases: similarities and differences in template usage between tombusvirus and carmovirus RNA-dependent RNA polymerases.. Virology.

[39] Pogany J, Fabian MR, White KA, Nagy PD (2003). A replication silencer element in a plus-strand RNA virus.. Embo J.

[40] Pathak KB, Nagy PD (2009). Defective Interfering RNAs: Foes of Viruses and Friends of Virologists.. Viruses-Basel.

[41] White KA, Nagy PD (2004). Advances in the molecular biology of tombusviruses: gene expression, genome replication, and recombination.. Prog Nucleic Acid Res Mol Biol.

[42] Jonczyk M, Pathak KB, Sharma M, Nagy PD (2007). Exploiting alternative subcellular location for replication: tombusvirus replication switches to the endoplasmic reticulum in the absence of peroxisomes.. Virology.

[43] Panavas T, Hawkins CM, Panaviene Z, Nagy PD (2005). The role of the p33:p33/p92 interaction domain in RNA replication and intracellular localization of p33 and p92 proteins of Cucumber necrosis tombusvirus.. Virology.

[44] Johnson CM, Perez DR, French R, Merrick WC, Donis RO (2001). The NS5A protein of bovine viral diarrhoea virus interacts with the alpha subunit of translation elongation factor-1.. J Gen Virol.

[45] Qanungo KR, Shaji D, Mathur M, Banerjee AK (2004). Two RNA polymerase complexes from vesicular stomatitis virus-infected cells that carry out transcription and replication of genome RNA.. Proc Natl Acad Sci U S A.

[46] Yamaji Y, Sakurai K, Hamada K, Komatsu K, Ozeki J (2010). Significance of eukaryotic translation elongation factor 1A in tobacco mosaic virus infection.. Arch Virol.

[47] Gavin AC, Bosche M, Krause R, Grandi P, Marzioch M (2002). Functional organization of the yeast proteome by systematic analysis of protein complexes.. Nature.

[48] Gavin AC, Aloy P, Grandi P, Krause R, Boesche M (2006). Proteome survey reveals modularity of the yeast cell machinery.. Nature.

[49] Collins SR, Kemmeren P, Zhao XC, Greenblatt JF, Spencer F (2007). Toward a comprehensive atlas of the physical interactome of Saccharomyces cerevisiae.. Mol Cell Proteomics.

[50] Panavas T, Pogany J, Nagy PD (2002). Analysis of minimal promoter sequences for plus-strand synthesis by the Cucumber necrosis virus RNA-dependent RNA polymerase.. Virology.

[51] Al-Maghrebi M, Brule H, Padkina M, Allen C, Holmes WM (2002). The 3′ untranslated region of human vimentin mRNA interacts with protein complexes containing eEF-1gamma and HAX-1.. Nucleic Acids Res.

[52] Blumenthal T, Young RA, Brown S (1976). Function and structure in phage Qbeta RNA replicase. Association of EF-Tu-Ts with the other enzyme subunits.. J Biol Chem.

[53] Blumenthal T, Landers TA, Weber K (1972). Bacteriophage Q replicase contains the protein biosynthesis elongation factors EF Tu and EF Ts.. Proc Natl Acad Sci U S A.

[54] Dreher TW (1999). Functions of the 3′-Untranslated Regions of Positive Strand Rna Viral Genomes.. Annu Rev Phytopathol.

[55] Thivierge K, Cotton S, Dufresne PJ, Mathieu I, Beauchemin C (2008). Eukaryotic elongation factor 1A interacts with Turnip mosaic virus RNA-dependent RNA polymerase and VPg-Pro in virus-induced vesicles.. Virology.

[56] Nishikiori M, Dohi K, Mori M, Meshi T, Naito S (2006). Membrane-bound tomato mosaic virus replication proteins participate in RNA synthesis and are associated with host proteins in a pattern distinct from those that are not membrane bound.. J Virol.

[57] Zeenko VV, Ryabova LA, Spirin AS, Rothnie HM, Hess D (2002). Eukaryotic elongation factor 1A interacts with the upstream pseudoknot domain in the 3′ untranslated region of tobacco mosaic virus RNA.. J Virol.

[58] De Nova-Ocampo M, Villegas-Sepulveda N, del Angel RM (2002). Translation elongation factor-1alpha, La, and PTB interact with the 3′ untranslated region of dengue 4 virus RNA.. Virology.

[59] Bastin M, Hall TC (1976). Interaction of elongation factor 1 with aminoacylated brome mosaic virus and tRNA's.. J Virol.

[60] Sikora D, Greco-Stewart VS, Miron P, Pelchat M (2009). The hepatitis delta virus RNA genome interacts with eEF1A1, p54(nrb), hnRNP-L, GAPDH and ASF/SF2.. Virology.

[61] Dube A, Bisaillon M, Perreault JP (2009). Identification of proteins from prunus persica that interact with peach latent mosaic viroid.. J Virol.

[62] Jeppesen MG, Ortiz P, Shepard W, Kinzy TG, Nyborg J (2003). The crystal structure of the glutathione S-transferase-like domain of elongation factor 1Bgamma from Saccharomyces cerevisiae.. J Biol Chem.

[63] Nagy PD, Carpenter CD, Simon AE (1997). A novel 3′-end repair mechanism in an RNA virus.. Proc Natl Acad Sci U S A.

[64] Zhang Y, Nijbroek G, Sullivan ML, McCracken AA, Watkins SC (2001). Hsp70 molecular chaperone facilitates endoplasmic reticulum-associated protein degradation of cystic fibrosis transmembrane conductance regulator in yeast.. Mol Biol Cell.

[65] Jaag HM, Nagy PD (2009). Silencing of Nicotiana benthamiana Xrn4p exoribonuclease promotes tombusvirus RNA accumulation and recombination.. Virology.

[66] Jaag HM, Pogany J, Nagy PD (2010). A host Ca2+/Mn2+ ion pump is a factor in the emergence of viral RNA recombinants.. Cell Host Microbe.

